# Supplementary material for: Macrophage Migration Inhibitory Factor on Apoptotic Extracellular Vesicles Regulates Compensatory Proliferation
Source: bioRxiv. 2023 Jun 14:2023.06.14.544889. Preprint. [Version 1] doi: 10.1101/2023.06.14.544889 (PMC10312732; doi:10.1101/2023.06.14.544889)

bioRxiv preprint doi: https://doi.org/10.1101/2023.06.14.544889; this version posted June 14, 2023. The copyright holder for this preprint (which was not certified by peer review) is the author/funder, who has granted bioRxiv a license to display the preprint in perpetuity. It is made available under aCC-BY 4.0 International license.

SUPPLEMENTAL FIGURE LEGENDS

Supplemental Figure 1. D-DT (MIF-2) is not localized to the surfaces of esAEVs. (A) A representative image of an esAEV administered a control stain of Secondary Alone (also referred to as secondary alone). Size bar = 200 nm. (B) D-DT staining on an esAEV. Scale bar = 400 nm (C) Annexin staining on esAEV. n = 200 nm. (D) Quantitative representation of nanogold particles for Secondary Alone (mean+/-SEM = 0.000 +/- 0.000), D-DT (mean = 4.350 +/- 0.8438), and Annexin V (mean = 33.00 +/- 5.310). Each dot represents an individual esAEV as represented in panels A-C. n = 18, Secondary Alone. n = 24, Annexin V. n = 20, D-DT. The difference between means for Annexin V and D-DT was significant (adjusted p-value<0.0001). There was not a statistically significant difference in means between D-DT and Secondary alone (p = 0.6939).

Supplemental Figure 2. Genotyping of F0 CRISPR-edited larvae using sanger sequencing and fluorescent *in situ* hybridization (FISH). (A, E, I) The gene structures for *mif*, *cd74a*, and *cd74b*. The sgRNAs for each gene are represented along with the general locations within each respective gene. The green text highlights the PAM sequences. The text in the parenthesis are the ensembl genome browser accession numbers, and the information that was entered into CHOP-CHOP to design sgRNAs. (B, F, J) FISH staining of uninjected vs *mif*, *cd74a*, and *cd74b* CRISPR larvae respectively. (C, G, K) The comparison CRISPR editing for two cut sites for *mif*, *cd74a*, and *cd74b*. Magenta represents cutting efficiencies greater than 50%, and black represent less than 50% cutting efficiency as predicted by TIDE or ICE analysis. R-squared values represent the sequence alignment between control and CRISPR-edited samples. (D, H, L) Representative signal traces comparing control to CRISPR edited larvae upstream of the PAM site for *mif* (B), *cd74a* (F), and *cd74b* (J). Scale bars = 50 μm.

Supplemental Figure 3. CRISPR/Cas9 editing of *tyrosinase* does not affect apoptosis-induced proliferation. (A) Phenotypic differences between no injection and CRISPR-edited *tyrosinase* larvae. The loss of pigment is a visual confirmation of successful *tyrosinase* gene editing, and this serves as a proxy for the functionality of the Cas9 protein. (B) Assessment of apoptosis-induced proliferation between uninjected larvae and *tyrosinase* CRISPR larvae after the addition of MTZ. A two-way ANOVA demonstrates that there is no statistical difference between the means for MTZ-treated uninjected and *tyrosinase* CRISPR. n = 41, uninjected,

bioRxiv preprint doi: https://doi.org/10.1101/2023.06.14.544889; this version posted June 14, 2023. The copyright holder for this preprint (which was not certified by peer review) is the author/funder, who has granted bioRxiv a license to display the preprint in perpetuity. It is made available under aCC-BY 4.0 International license.

DMSO. n = 59 uninjected, MTZ. n = 23 *tyrosinase* CRISPR, DMSO. n = 40 *tyrosinase* CRISPR, MTZ. Scale bar = 1000 μm.

Supplemental Figure 4. *cd74a* and *cd74b* expression in macrophages. (A) A representative image of macrophage location during homeostatic conditions in an *mpeg:GFP* transgenic line. (B) A representative image of *cd74a* transcripts in macrophages (B') at and away from the amputation site. (C) A representative image of *cd74b* transcripts in macrophages (C') at and away from the amputation site. Scale bars: A, B, and C = 50 μm, B' and C' = 5 μm.

Supplemental Figure 5. Heat-shock induction of MIF-GFP does not induce proliferation under homeostatic conditions. (A) Schema of the Tol2 construct used to drive *mif-gfp* downstream of the hsp70 promoter. Encoded within the genetic construct is a green heart marker using *cmlc2:gfp* to initially pick select larvae with the construct. All constructs were co-injected with transposase mRNA. **(B)** Representative large-field images of a clutch of zebrafish larvae before and after heat-shock induction of MIF-GFP. **(C)** A 10x confocal image of the distribution of MIF-GFP in a larvae pre and post heat shock induction. (D) A 20x confocal image of the variety of cell types within the tail epithelium expressing MIF-GFP. **(E)** A comparison of proliferation in heat-shocked and non-heat shocked larvae. n = 36, no inj. n = 32, heat shock. A student's t-test was used to assess differences in means. Scale bars: B = 100 μm, C = 500 μm, D = 50 μm.

Supplemental Figure 6. Treatment with secretion inhibitors do not alter apoptosis-induced proliferation. (A)Treatment with Brefeldin A does not affect AEV formation. (B) Treatment with various concentrations of Brefeldin A does not induce a significant reduction in proliferation. (C) esAEV formation after treatment with glyburide. (D) Apoptosis-induced proliferation in the presence of glyburide. Each condition had 32-40 larvae. Statistical significance was calculated with a Two-way ANOVA with a Tukey's post hoc test. Scales bars = 50 μm.

Supplemental Figure 7. p-ERK signaling was not detectable in macrophages. (A) Representative images of p-ERK signaling in an *mpeg:GFP* background across three different timepoints post-MTZ treatment. (B) Images of 3 ROIS selected per timepoint highlighting the p-ERK level in macrophages. Scale bar = 50 μm.

bioRxiv preprint doi: https://doi.org/10.1101/2023.06.14.544889; this version posted June 14, 2023. The copyright holder for this preprint (which was not certified by peer review) is the author/funder, who has granted bioRxiv a license to display the preprint in perpetuity. It is made available under aCC-BY 4.0 International license.

Supplemental Figure 8. Macrophage contribution to AEV engulfment and depletion with *irf8* morpholino. (A)The number of epithelial stem cells that undergo apoptosis up to 3hrs post treatment. n = 5 larvae, DMSO. n = 6, MTZ. **** <0.0001 using a two-tailed t-test. (B) The number of engulfment events of macrophages in the presence of apoptotic cells across an 8-hour timespan. n = 8 larvae, DMSO. n = 6, MTZ. *** 0.0003 using a two-tailed t-test. (C) Ablation of the macrophage lineage using *irf8* morpholino (scale bar =200 μm). (D) Quantifications of the number of macrophages at 3dpf. n= 72 for Wild-type and n = 53 for *irf8* morpholino. ****<0.0001 via an unpaired two-tailed test. (E) Quantifications of the number of macrophages at 4dpf. n = 31, Wild-type. n = 21, *ifr8* morpholino. ** 0.0029 using an unpaired two-tailed t-test. Quantifications for D,E were performed by counting the number of macrophages present in the caudal vein from the cloaca to the tip of the notochord. Scale bar = 200 μm.

SUPPLEMENTAL TABLES

Table 1. Proteins identified in the proteomic analysis of esAEVs.

SUPPLEMENTAL MOVIES

Supplemental Movie 1: The formation of esAEVs. Time-lapse imaging of NTR-mCherry cells undergoing apoptosis and forming esAEVs *in vivo*. Images were acquired every 5 minutes across a z-stack and compiled as a maximum intensity projection, 2.5 fps. Scale bar = 10 μm. Time = hh:mm:ss.

Supplemental Movie 2: Epithelial stem cell engulfment of apoptotic extracellular vesicles. Time-lapse imaging of a p63:GFP positive cell engulfing an NTR-positive esAEV. Images were acquired every 15 minutes across a z-stack for 5 hours. 2.5 fps. Scale bar = 25 μm. Time = hh:mm:ss.

Supplemental Movie 3: Macrophage dynamics during homeostasis and after induced apoptosis. Time-lapse imaging of macrophage dynamics in an NTR-mCherry background. (Left) Macrophage movement with DMSO treatment. (Right) Macrophage movement after 4 hours of MTZ treatment. Images were acquired every 5 minutes across a z-stack for 8 hours. 5.0 fps. Scale bar = 50 μm. Time = hh:mm:ss.

bioRxiv preprint doi: https://doi.org/10.1101/2023.06.14.544889; this version posted June 14, 2023. The copyright holder for this preprint (which was not certified by peer review) is the author/funder, who has granted bioRxiv a license to display the preprint in perpetuity. It is made available under aCC-BY 4.0 International license.

Supplemental Figure 1

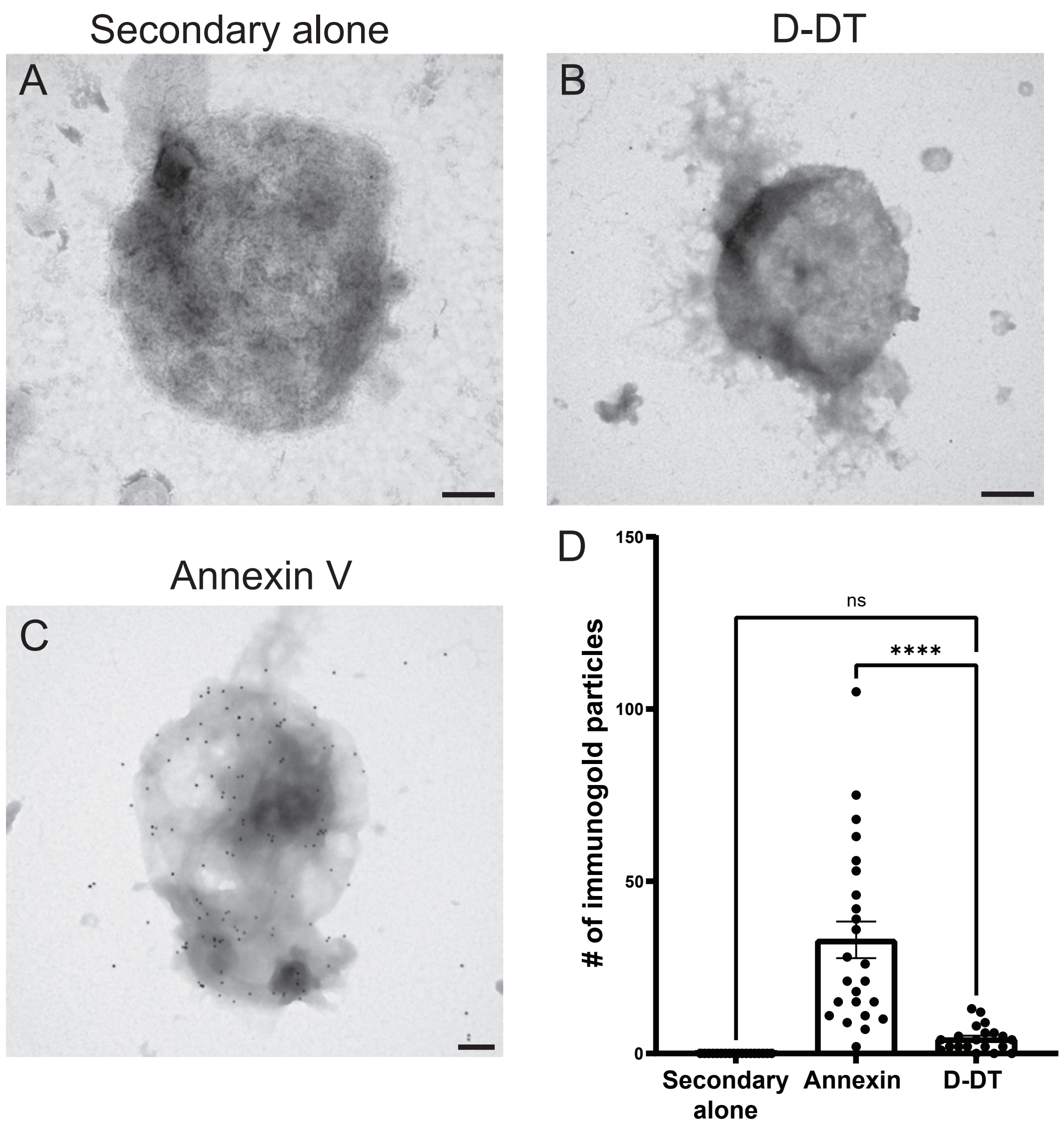

bioRxiv preprint doi: https://doi.org/10.1101/2023.06.14.544889; this version posted June 14, 2023. The copyright holder for this preprint (which was not certified by peer review) is the author/funder, who has granted bioRxiv a license to display the preprint in perpetuity. It is made available under aCC-BY 4.0 International license.

Supplemental Figure 2

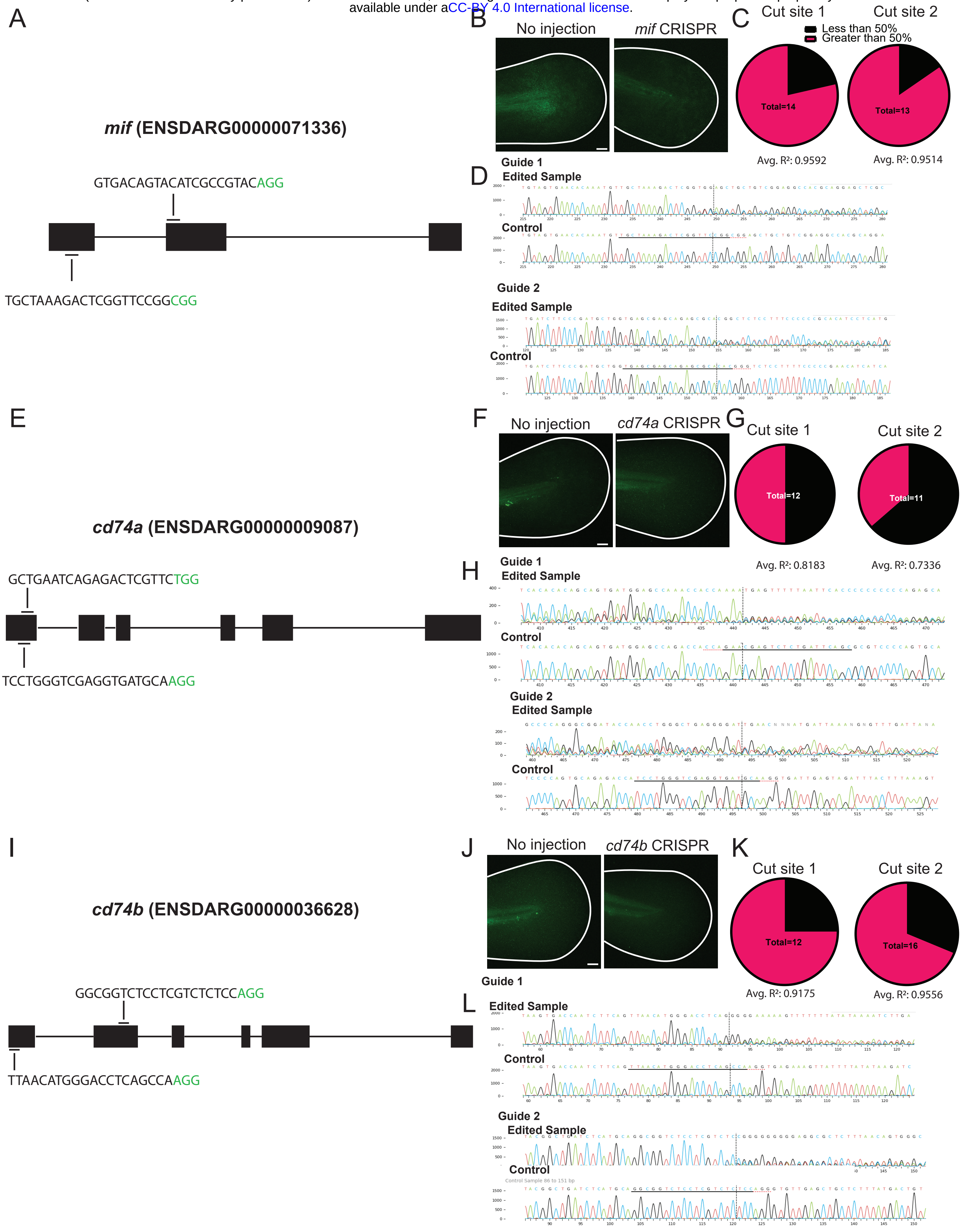

bioRxiv preprint doi: https://doi.org/10.1101/2023.06.14.544889; this version posted June 14, 2023. The copyright holder for this preprint (which was not certified by peer review) is the author/funder, who has granted bioRxiv a license to display the preprint in perpetuity. It is made available under aCC-BY 4.0 International license.

Supplemental Figure 3

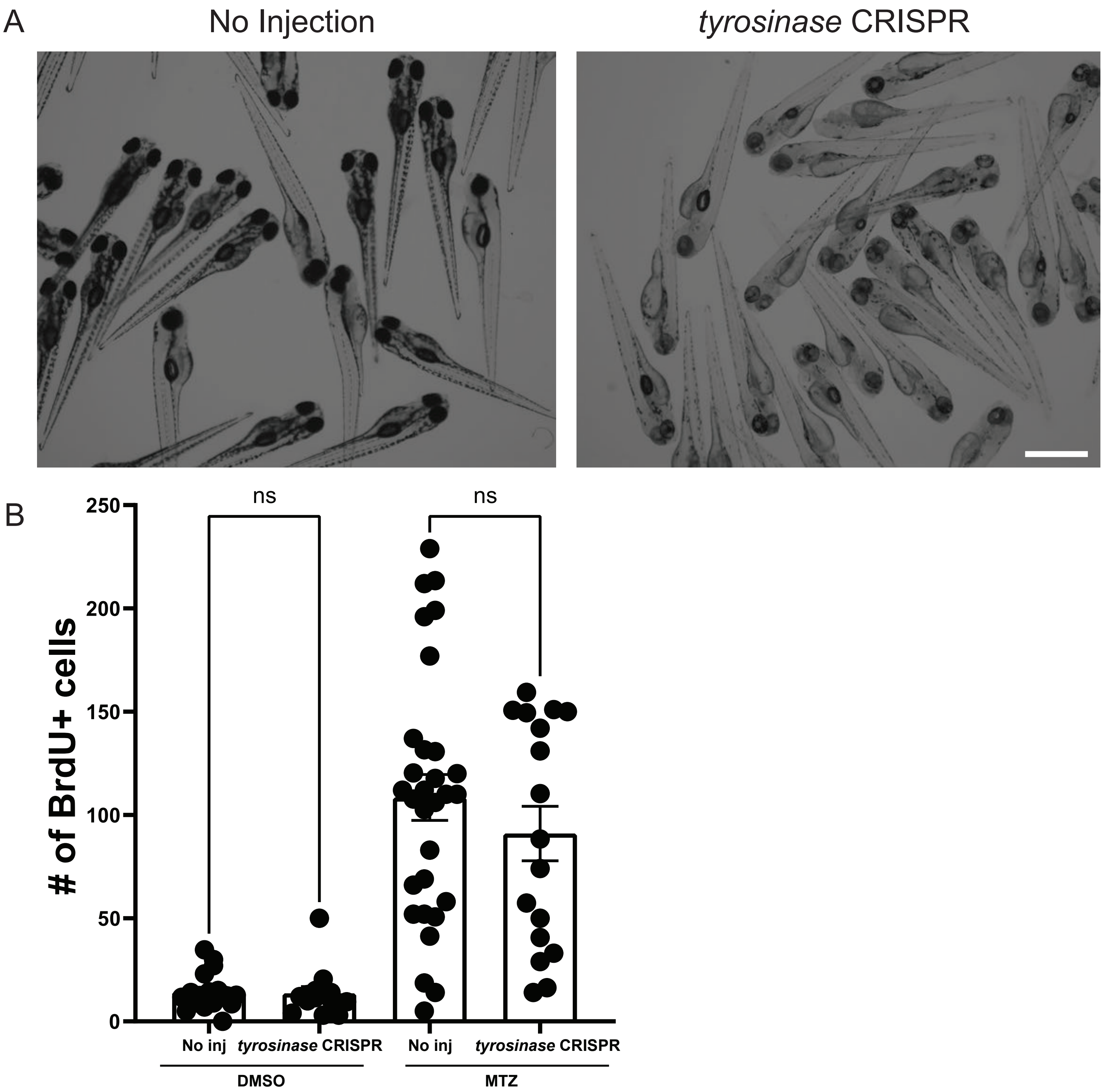

bioRxiv preprint doi: https://doi.org/10.1101/2023.06.14.544889; this version posted June 14, 2023. The copyright holder for this preprint (which was not certified by peer review) is the author/funder, who has granted bioRxiv a license to display the preprint in perpetuity. It is made available under aCC-BY 4.0 International license.

Supplemental Figure 4

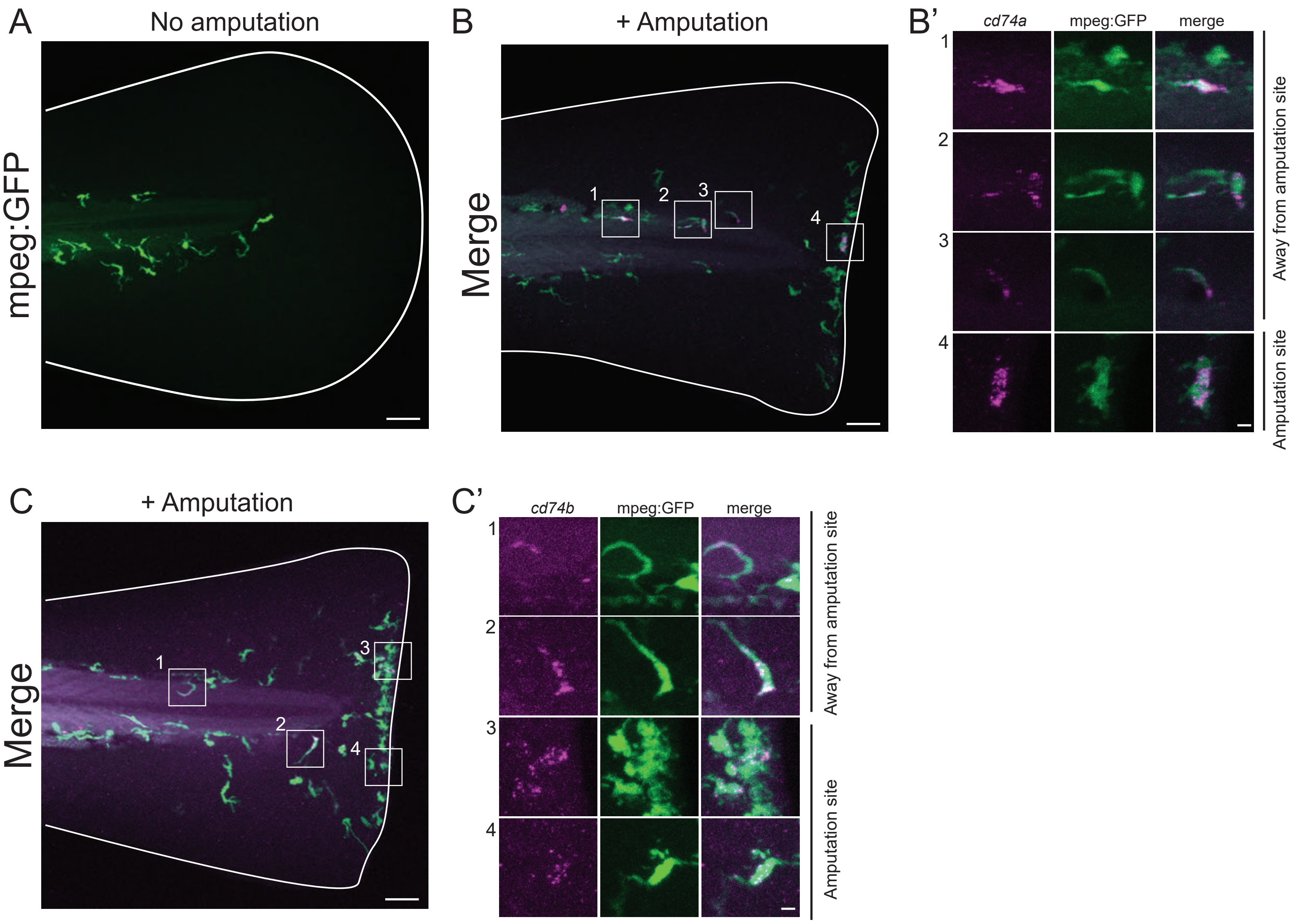

bioRxiv preprint doi: https://doi.org/10.1101/2023.06.14.544889; this version posted June 14, 2023. The copyright holder for this preprint (which was not certified by peer review) is the author/funder, who has granted bioRxiv a license to display the preprint in perpetuity. It is made available under aCC-BY 4.0 International license.

Supplemental Figure 5

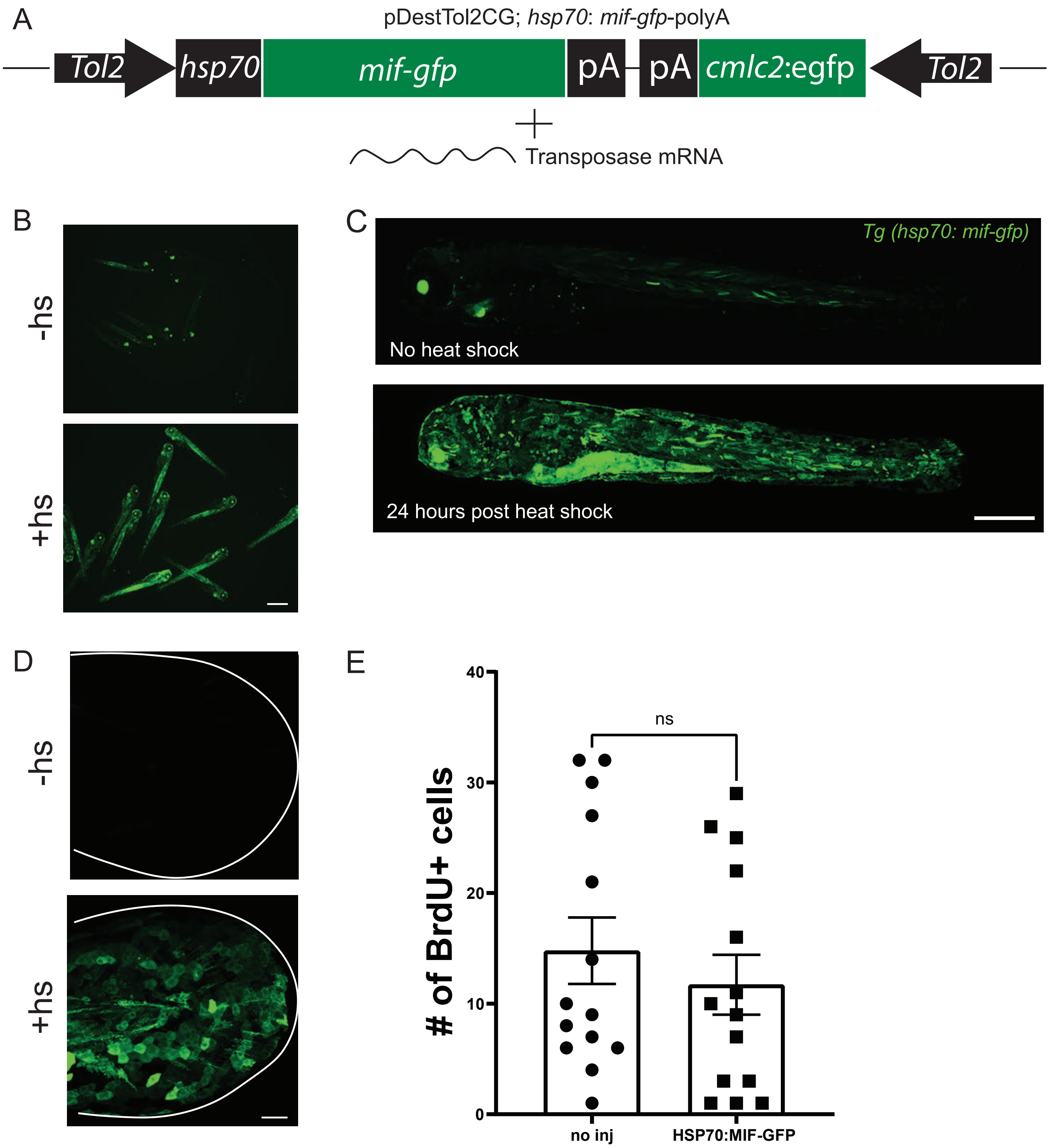

bioRxiv preprint doi: https://doi.org/10.1101/2023.06.14.544889; this version posted June 14, 2023. The copyright holder for this preprint (which was not certified by peer review) is the author/funder, who has granted bioRxiv a license to display the preprint in perpetuity. It is made available under aCC-BY 4.0 International license.

Supplemental Figure 6

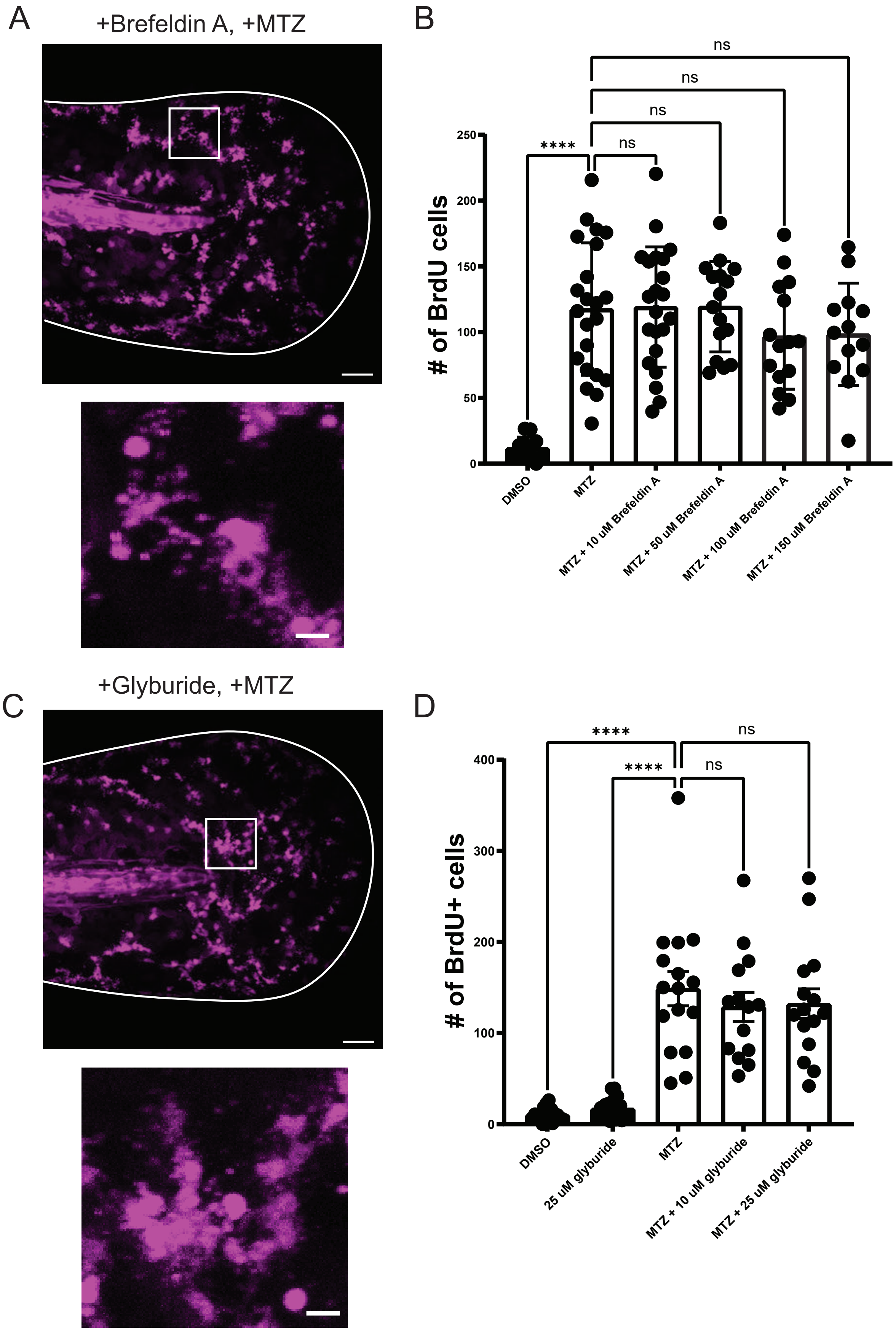

bioRxiv preprint doi: https://doi.org/10.1101/2023.06.14.544889; this version posted June 14, 2023. The copyright holder for this preprint (which was not certified by peer review) is the author/funder, who has granted bioRxiv a license to display the preprint in perpetuity. It is made available under aCC-BY 4.0 International license.

Supplemental Figure 7

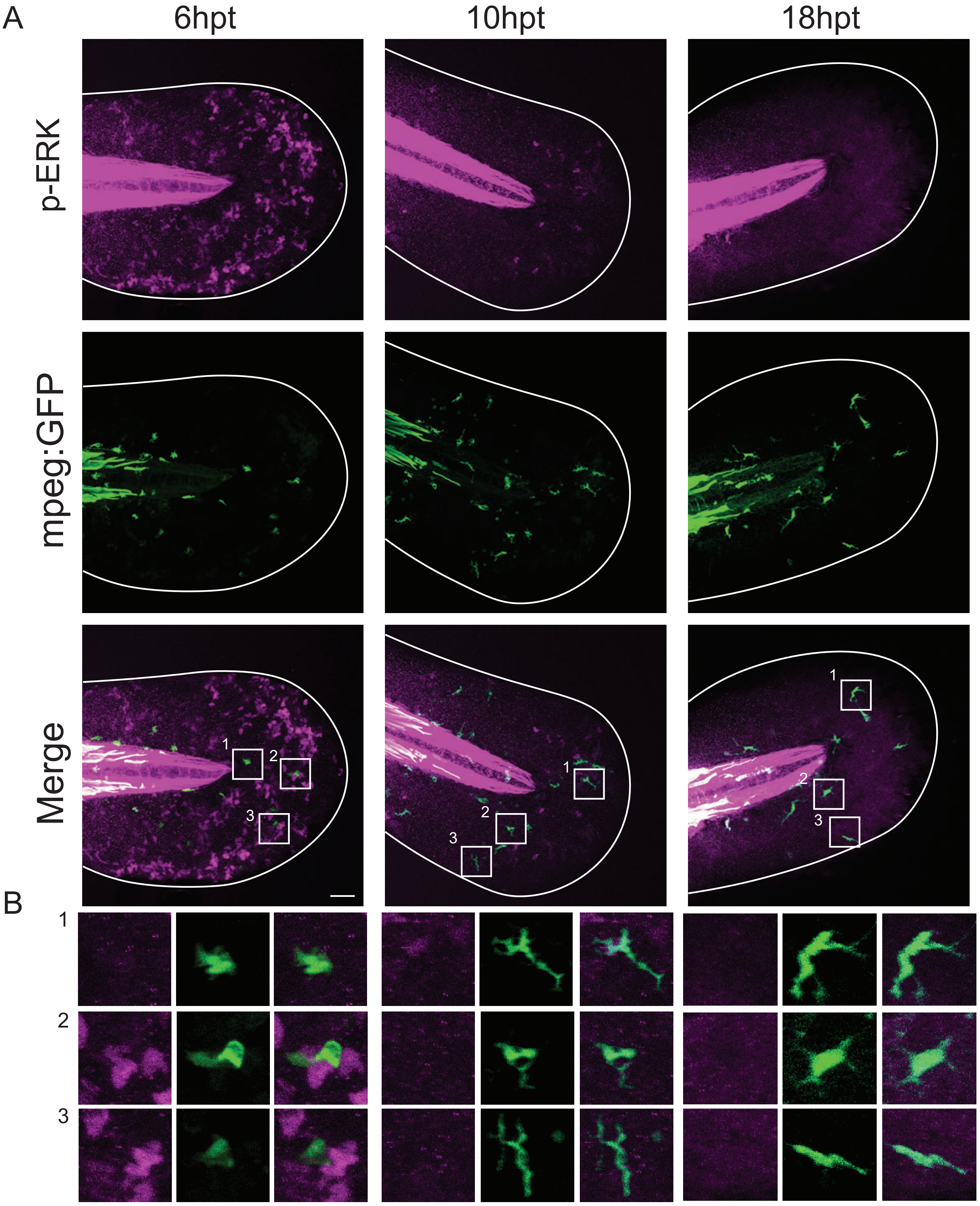

bioRxiv preprint doi: https://doi.org/10.1101/2023.06.14.544889; this version posted June 14, 2023. The copyright holder for this preprint (which was not certified by peer review) is the author/funder, who has granted bioRxiv a license to display the preprint in perpetuity. It is made available under aCC-BY 4.0 International license.

Supplemental Figure 8

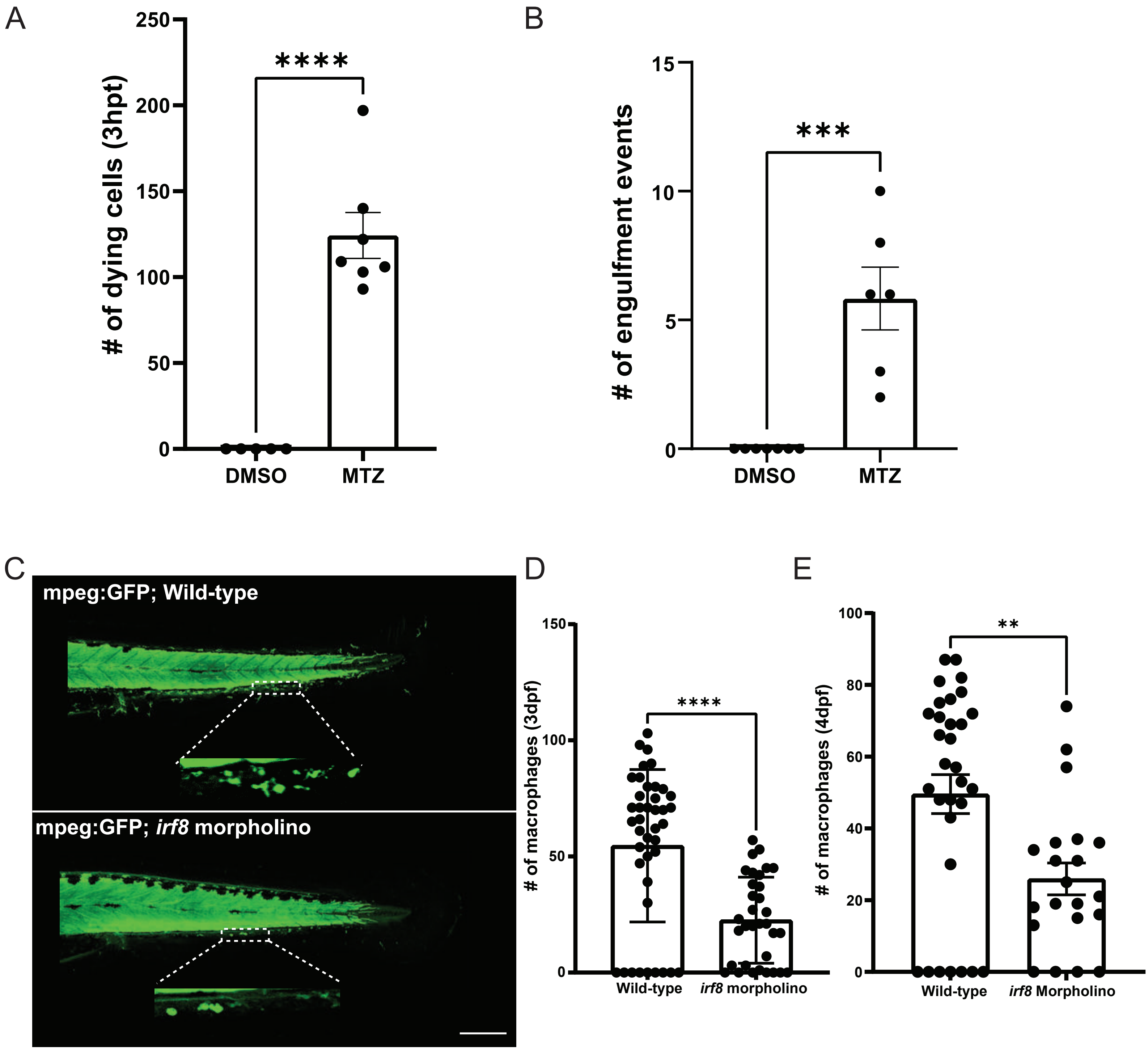

Supplement: 1 [file NIHPP2023.06.14.544889v1-supplement-1.pdf]
